# The role of LCCL proteins in malaria transmission

**DOI:** 10.1186/1475-2875-9-S2-O27

**Published:** 2010-10-20

**Authors:** Sadia Saeed, Annie Z Tremp, Johannes T Dessens

**Affiliations:** 1 Department of Pathogen Molecular Biology, London School of Hygiene &Tropical Medicine, Keppel Street, London WC1E 7HT, UK

## Background

Malaria is transmitted by sporozoite-infected mosquito bites. Due to their key role in transmission, sporozoites are an important target for malaria control strategies. The *Plasmodium* LCCL protein family has six members, which share a modular structure composed of multiple domains implicated in protein, lipid and carbohydrate binding, they have essential roles in sporozoite development and infectivity. The type member of this family in *P. berghei,* named PbSR or PbLAP1, is synthesized in macrogametocytes, gets trafficked to the ookinete's crystalloid organelles, and is essential for crystalloid organelle formation [1]. Similar gametocyte-specific expression and loss-of-function phenotypes have been reported for other LCCL protein family members, suggesting that they may be involved in a common molecular mechanism and may be operating in concert, possibly as a protein complex. In this study we investigate the expression, sub cellular localisation, trafficking and function of PbLAP3, the last member of the LCCL protein family to be functionally characterised in *P. berghei.*

## Method

To study the expression and localization, genetically modified parasite lines were generated expressing PbLAP3 tagged with green fluorescent protein (GFP) at the C-terminus. This was achieved by the replacement of native *PbLAP3* gene by GFP-tagged genes via double crossover homologous recombination. Life stage expression in ookinete was studied by setting up ookinete culture, expression in oocyst and sporozoite was assessed by mosquito infection. Live parasites were observed by confocal and UV microscopy. Localization of the PbLAP3 was studied by immune gold EM.

## Results

**Figure 1:**
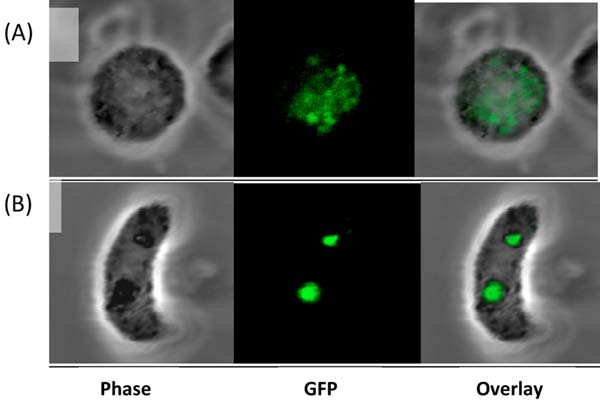
Expression and subcellular localisation of GFP-tagged PbLAP3 in gametocytes and ookinetes of *Plasmodium berghei.*

Analysis of GFP fluorescence of PbLAP3/EGFP by confocal microscopy revealed PbLAP3 expression in gametocytes (Figure 1a) [2]. In ookinetes GFP fluorescence was observed as two cytoplasmic focal spots (Figure 1b) [2]. These spots are characteristic for the crystalloids [1]. Green fluorescence was not detected in oocysts, nor in sporozoites of parasite line PbLAP3/EGFP, which indicate that PbLAP3 is not expressed in these life stages [2].

## Conclusion

LCCL proteins play a central role in the function of crystalloids in *P. berghei.* The new cellular markers for the crystalloids identified here will provide useful new tools for addressing intriguing questions about the molecular processes that lead to crystalloids formation which then facilitate the sporozoite development.
